# A Novel Approach to Ileal Pouch Prolapse Repair Using Fibrin Sealant

**DOI:** 10.7759/cureus.28264

**Published:** 2022-08-22

**Authors:** Christina Provenza, Constantine Poulos, Rachel Scott, Saumitra Banerjee

**Affiliations:** 1 Surgery, University of Connecticut School of Medicine, Farmington, USA; 2 Surgery, Colon and Rectal Surgeons of Greater Hartford, Bloomfield, USA

**Keywords:** colorectal surgery, surgical case reports, ileal pouch prolapse repair method, fibrin sealant, ileal pouch prolapse

## Abstract

The ileal pouch-anal anastomosis is a commonly accepted neorectum after total proctocolectomy for familial adenomatous polyposis and ulcerative colitis. Generally, patients have decent bowel control, but ileal pouches are not without complications. One relatively uncommon complication is ileal pouch prolapse. Prolapse can be either mucosal or full thickness, similar to rectal prolapse. There is limited literature detailing the frequency and management of ileal pouch prolapse. The majority of the literature is case reports with a few small retrospective studies. Fibrin glue has been described for sutureless mesh fixation in total extraperitoneal hernia repairs. Here, we describe a fibrin glue pouch pexy for ileal pouch prolapse after total proctocolectomy with ileal pouch-anal anastomosis.

## Introduction

Ileal pouch prolapse is defined as a protrusion of the pouch wall into the lumen or through the anal canal [1]. Blazeby first described the condition in 1994, realizing ileal pouch prolapse is very similar to rectal prolapse. Since then, literature on ileal pouch prolapse management has only included a hand full of case reports [2-7] and series [8,9], with one retrospective survey study [10]. Ileal pouch prolapse is a rare complication, reportedly affecting 0.3-0.35% of all patients who undergo the procedure [8,10]. The average age has been reported at 34 years [8] and usually occurs within two years of surgery [10]. Unlike rectal prolapse, ileal pouch prolapse has no predilection for men or women [8], but one study found it to be more common in young females with low BMI and in patients with little peripouch fat [1].

Risk factors for ileal pouch prolapse are unknown; however, the onset could be related to low body weight and a family history of ulcerative colitis [11]. Symptoms include painful defecation, tissue protrusion, excessive straining, anal seepage, incomplete evacuation, nausea, bloating, and perianal dermatitis [1,8,10]. These symptoms can easily be incorrectly attributed to pouchitis, described by Blazeby as a possible misdiagnosis [8,12]. Diagnosis can be made clinically but is aided by pouchoscopy, pouch defecography, or pelvic MRI [1,3,8].

There are two primary categories of ileal pouch prolapse: mucosal prolapse and full-thickness prolapse. Mucosal prolapse is generally managed conservatively with a high fiber diet and biofeedback [1,8,13]. Refractory symptoms can be intervened upon with transanal excision or banding of prolapsed tissue [1]. One case report used an endoscopic hot snare to resect excess tissue [2]. Full-thickness prolapse must be managed surgically by fixation of the pouch to the sacral promontory, similar to rectopexy for rectal prolapse. However, there is a concern for the durability of the pouch in suture pexy [8]. Several fixation methods have been described in case reports including suture pexy alone to presacral fascia [3,8], pexy with biologic mesh [7-9], and pexy with permanent mesh [5,6]. One case report used a perineal approach to suspend the pouch to the pelvic side walls using collagen [4]. Transabdominal repairs have been performed as laparoscopic [7], robotic [6], and open [9].

In general surgical literature, fibrin sealant has been documented to provide adequate, sutureless fixation of mesh during total extraperitoneal hernia repairs [14-16]. When compared to traditional mechanical fixation, sutureless fibrin fixation of mesh had comparable recurrence rates at a mean follow-up of 1.2 years [14]. Additionally, patients with fibrin sealant fixation had reduced postoperative analgesic use, as well as significantly lower rates of chronic pain [14,15]. Similar sutureless approaches have been used in laparoscopic rectopexy in combination with mesh fixation [16]. With that said, these sutureless techniques have not been extended to the treatment of ileal pouch prolapse. Utilizing sutureless fixation of ileal pouch prolapse may be advantageous as the pouched small bowel lacks the durability of the rectum during traditional rectopexy. To our knowledge, this is the first description of a laparoscopic repair using fibrin glue pexy for ileal pouch prolapse after total proctocolectomy with ileal pouch-anal anastomosis.

## Case presentation

Our patient was a 56-year-old woman with a BMI of 23.2, who presented in April 2019, approximately 11 years status post total proctocolectomy with ileal J-pouch creation secondary to ulcerative colitis. The patient’s medical history was significant for diabetes, gastroesophageal reflux, hypertension, anxiety, fibromyalgia, migraines, and vulvodynia. Previous abdominal surgical history included a total abdominal hysterectomy and ventral hernia repair in addition to total proctocolectomy and ileostomy reversal. The patient’s initial complaints included increased bowel frequency, constant urge to defecate, and sensation of incomplete evacuation. She also had some blood per rectum, which she attributed to internal hemorrhoids.

**Figure 1:**
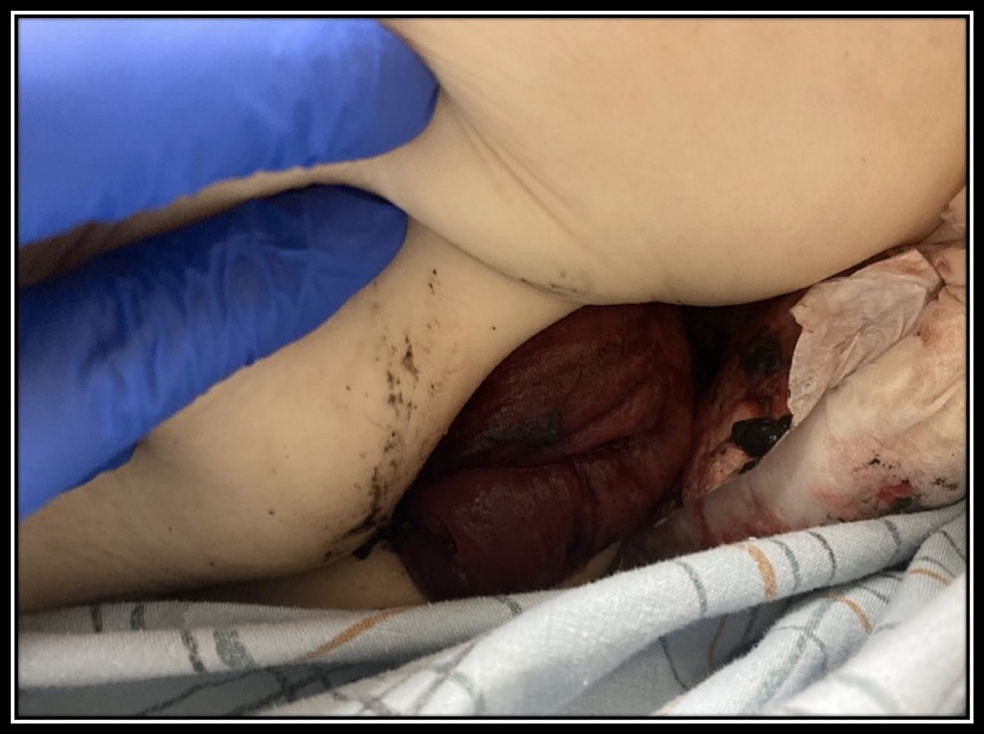
Ileal pouch prolapse prior to surgery

For these symptoms, she was seen by her primary care provider, who had previously ordered stool studies, which were negative. She had never had a pouchoscopy but was treated for presumed pouchitis. After treatment, her symptoms persisted. A physical exam showed no significant hemorrhoids and a normal digital rectal exam. Two months after the initial presentation in June of 2019, she had an episode of complete pouch prolapse requiring surgical reduction under anesthesia (Figure 1). Upon follow-up, she continued to have symptoms of intermittent prolapse.

She was deemed an appropriate candidate for elective surgical management. She was taken to the operating room and placed under general anesthesia. Entry to the abdomen was achieved with Hasson port, and immediately extensive adhesions were encountered. A hand port was placed to facilitate intra-abdominal lysis of adhesions. Once the pelvis was accessed, the presacral area was freed of adhesions, and the pouch mobilized cephalad. The patient had significant pelvic floor laxity. Traditional suture rectopexy was not performed due to fear of enterotomy to the ileal pouch.

Kit Evicel 2 ml Sealant Fibrin tissue closure (Ethicon Endo-Surgery, Cincinnati, Ohio) was applied posteriorly to the pouch and on the left side of the pelvis. The pressure was held for five minutes to ensure a good seal. The pouch did not appear to move during the release of the pneumoperitoneum. The patient tolerated the procedure well and was admitted to the floor postoperatively.

Her hospital course was complicated by worsened baseline vertigo and urinary retention. Bowel function returned on postoperative day two, and she was ultimately discharged on postoperative day five. Since surgery, bowel regularity has been maintained with a high fiber diet and fiber supplementation. She remained symptom-free but unfortunately recurred after 13 months. She had multiple episodes of prolapse that required a reduction in the emergency room. She has not undergone any further surgical repair. The long-term efficacy of fibrin glue for ileal pouch prolapse repair is still under investigation.

## Discussion

Ileal pouch prolapse is a rare complication following ileal J-pouch creation, occurring in approximately 0.3-0.35% of cases [8,10]. With recurrent symptoms of frequency, tenesmus, and rectal pain, mild cases of ileal pouch prolapse are often misdiagnosed as pouchitis. Full thickness, irreducible prolapse of the ileal pouch warrants surgical intervention.

As expected, transabdominal approaches to ileal pouch prolapse are similar to laparoscopic rectopexy [8]. However, given the lack of a durable rectum, extra care must be taken during the reduction and fixation of ileal pouches. Repairs with both biologic and permanent meshes have been described, but are dangerous given the potential for full-thickness injury during fixation [5-9]. Mesh placement in this patient was deferred given the recurrent nature of this patient’s ileal pouch prolapse and the potential need for future revision.

Therefore, we opted for fixation with fibrin glue as an alternative modality for pouch pexy. Fibrin sealant has been studied in hernia literature as a means for temporary fixation. For cases of ileal pouch prolapse, fibrin sealant may provide temporary but durable fixation to allow adhesive fixation of the ileal pouch. While our repair has yet to prove durable in long-term follow-up, short-term resolution of symptoms has been promising. Fibrin fixation may be useful in high-risk patients to avoid more extensive intervention including extensive pouch mobilization or takedown and ileostomy creation.

## Conclusions

In conclusion, ileal pouch prolapse is an uncommon complication after total proctocolectomy with an ileal pouch. Fibrin glue is an alternative to mesh or suture pexy to repair ileal pouch prolapse. The long-term durability of such a repair is still to be seen.
